# Comparison of Mesenchymal Stem Cell Surface Markers from Bone Marrow Aspirates and Adipose Stromal Vascular Fraction Sites

**DOI:** 10.3389/fvets.2015.00082

**Published:** 2016-01-15

**Authors:** Meghan O. Sullivan, Wanda J. Gordon-Evans, Lisa Page Fredericks, Kristina Kiefer, Michael G. Conzemius, Dominique J. Griffon

**Affiliations:** ^1^Angell Animal Medical Center, Boston, MA, USA; ^2^Wisconsin Veterinary Referral Center, Waukesha, WI, USA; ^3^Department of Entomology, University of Illinois at Urbana–Champaign, Urbana, IL, USA; ^4^St. Paul Department of Veterinary Clinical Sciences, University of Minnesota, Minneapolis, MN, USA; ^5^College of Veterinary Medicine, Western University of Health Sciences, Pomona, CA, USA

**Keywords:** canine, adipose, bone marrow, stromal vascular fraction, mesenchymal stem cell

## Abstract

The objective of this study was to subjectively evaluate the harvest of two areas of adipose collection and three areas of bone marrow collection as potential sites for clinical harvest of adipose stromal vascular fraction (SVF) and bone marrow concentrate for clinical use by quantifying the amount of tissue harvested, subjective ease of harvest, the variation of each site, and determining the cell surface marker characteristics using commercially available antibodies. Bone marrow and adipose tissue samples were collected from 10 adult mixed breed dogs. Adipose tissue was collected from the caudal scapular region and falciform fat ligament. Bone marrow aspirates were collected from the ilium, humerus, and tibia. Tissues were weighed (adipose) or measured by volume (bone marrow), processed to isolate the SVF or bone marrow concentrate, and flow cytometry was performed to quantitate the percentage of cells that were CD90, CD44 positive, and CD45 negative. Sites and tissue types were compared using matched pairs *t*-test. Subjectively subcutaneous fat collection was the most difficult and large amounts of tissue dissection were necessary. Additionally the subcutaneous area yielded less than the goal amount of tissue. The bone marrow harvest ranged from 10 to 27.5 ml. Adipose tissue had the highest concentration of cells with CD90^+^, CD44^+^, and CD45^−^ markers (*P* < 0.05), and bone marrow had the highest total number of these cells at harvest (*P* < 0.05). Variation was high for all sites, but the adipose collection yielded more consistent results. These results describe the relative cellular components in the SVF of adipose tissue and bone marrow as defined by the biomarkers chosen. Although bone marrow yielded higher absolute cell numbers on average, adipose tissue yielded more consistent results. Fat from the falciform ligament was easily obtained with less dissection and therefore created less perceived relative patient trauma.

## Introduction

Mesenchymal stem cells (MSCs) are a population of cells, which have the ability for self-renewal, long-term viability, and multilineage potential (1). MSCs have been shown to have the ability to regenerate into bone, cartilage, muscle, tendon, ligament, adipose, and stroma (2). There has been increasing interest in the clinical use of MSCs in both human and animal research. MSCs can be isolated from multiple tissues and delivered in multiple forms. After harvest, cells may be cultured creating a more homogenous and biomarkers change over time and may be dependent on culture conditions (3–5).

However, in small animal veterinary medicine, cultured cells have been less commonly used. Instead, a heterogeneous cell population is concentrated from bone marrow aspirates or adipose tissue harvest prior to the injection back into the animal. An adipose-derived population of cells called stromal vascular fraction (SVF) is created by digesting the fat and concentrating the remaining cells. These mixed cell populations and some MSC-concentrated preparations have mainly been applied to enhance tendon and ligament repair, fracture healing and to manage joint diseases (2, 6–11).

The use of stem cells has gained popularity in veterinary clinics (2, 12) in many forms and now extends beyond orthopedics, to manage other inflammatory conditions, such as inflammatory bowel disease and acute thoracolumbar disk disease (13, 14).

In spite of this expansion, the SVF has not been characterized in dogs compared to humans or bone marrow concentrate (6, 15). The additional elements of preparations that have not been cultured likely include smooth muscle cells, pericytes, fibroblasts, endothelial cells, hematopoietic stem cells, leukocytes, and endothelial progenitor cells (4, 16). Autologous SVFs have been used commercially since 2003 in veterinary medicine without the knowledge of the cellular make-up of the treatment (2, 8). The justification of their clinical use was based on the assumption that the SVF in dogs would be similar to that in man.

Variation among tissue types and/or sites used for cell harvest may make one tissue or site more clinically advantageous. The benefits of collection of adipose tissue for cell isolation include more abundant tissue, accessibility, and potentially higher MSC populations (17). It is believed that adipose tissue has minimal donor site morbidity as compared with bone marrow harvesting (18). Adipose vascular fraction provides a rich source of adipose-derived mesenchymal stem cells (aMSCs) with one study finding 500 times more stem cells in adipose compared to bone marrow (19, 20). The frequency of aMSCs in humans is 300 times the MSC frequency in bone marrow, which is only 0.01–0.001% (2, 20). However, this may not be true in dogs.

Location of harvest may also affect the number of MSCs harvested. Studies have been performed in human research as well as other animal species to determine the collection site with the highest yield of MSCs, but this has not been performed in the canine despite the use of aMSCs clinically. A human study evaluating the effect of tissue-harvesting site on aMSC yield showed that proliferation or differentiation capacities was not dependent on the tissue-harvesting site, but yield depended on the site (21).

The objective of this study was twofold: (1) to subjectively evaluate and compare the difficulty of harvest of two areas of adipose collection and three areas of bone marrow collection. (2) To determine and compare the tissue yield, cellular yield, and variation of adipose SVF and bone marrow concentrate harvest for each site. First, we hypothesized that adipose tissue would be physically easier to harvest in adequate quantities than bone marrow. Second, we hypothesized that nucleated cell count would be higher in the adipose SVF when expressed as a percentage of tissue harvested. Third, we hypothesized that the falciform fat would yield a higher percentage of potential MSCs as defined by CD90^+^, CD44^+^, and CD45^−^ biomarkers.

## Materials and Methods

### Harvest

The samples were harvested from five sites antemortem with approval from the University of Minnesota Institutional Animal Care and Use Committee (approval number 0803A29646). Ten adult and mixed breed dogs being euthanized for other purposes were used for this study. Anesthesia was induced with propofol, the dogs were intubated, and maintained on isoflurane for tissue harvest. Dogs were humanely euthanized prior to anesthetic recovery with pentobarbital. Dogs were weighed and body condition score was assessed on a scale of 1–9. Bone marrow aspirates were aseptically harvested from the wing of the ilium, proximal medial tibia, and proximal humerus using an 11-gage Jamshedi needle similar to standard bone biopsy technique. The bone was palpated; a small incision was made into the skin and subcutaneous tissues. Once the needle was inserted, a 35-ml heparinized syringe (1000 IU/ml of blood) was used to aspirate bone marrow and blood.

Adipose tissue was aseptically harvested from the subcutaneous fat caudal to the scapula and the falciform fat ligament using standard surgical technique. The goal was to harvest 30 g at each site of adipose tissue collection. Caudal to the scapula, the skin was incised, and sharp and blunt dissection was used to remove the fat. For falciform fat harvest, a midline cranial abdominal incision was made and the falciform fat was collected with sharp dissection.

All fat samples were collected in 50 ml conical tubes and weighed. The adipose tissue was washed extensively with phosphate-buffered saline and minced. An equal volume of digest medium was added and shaken at 150 rpm at 37°C for 45 min. The digest medium was composed of Hank’s solution with 0.075% collagenase type 1, 1% bovine serum albumin, and 1% penicillin/streptomycin (PEST) solution (22, 23). The resulting solution was centrifuged at 250 × *g* for 5 min and the supernatant discarded. The bone marrow was treated with red cell lysis buffer, centrifuged at 250 × *g* for 5 min, and the supernatant was discarded.

For both adipose and bone marrow, the pellet was resuspended in Dulbecco’s Modified Eagle’s Medium with F-12 (DMEM/F-12) and filtered sequentially using 100 μm and 40 μm nylon mesh to remove cellular debris. The remaining cells were washed, counted using Trypan blue to distinguish viable from dead cells, and frozen in two million cells per milliliter aliquots. The freezing medium consisted of DMEM with 50% FBS and 10% dimethyl sulfoxide.

### Flow Cytometry

Prior to flow cytometry preparation, cells were thawed and placed in 10 ml of DMEM to dilute the dimethyl sulfoxide. The cells were collected by centrifugation at 400 × *g* for 5 min. Canine-specific or cross-reacting antibodies attached to fluorochromes [CD14 (Pacific Blue), CD34 (rPE), CD44 (APC-Cy7), CD45 (Alexa Fluor^®^ 647), and CD90 (PerCp-Cy5.5)] were purchased commercially.^1^ The APC-Cy7 and PerCp-Cy5.5 fluors were conjugated to their respective antibodies according to the manufacturer’s directions.^2^ Attachment of fluorescently labeled antibodies was done according to the manufacturer’s directions (see text footnote “a”). In brief, cells were incubated with the antibodies for at least 30 min at room temperature. Red cell lysis buffer was added, and samples were gently rocked in the dark for 10 min to eliminate the red cell population. After centrifugation at 400 × *g* for 5 min, cells were washed and resuspended in PBS and stored on ice until flow cytometry was performed. Flow cytometry was performed using a BD LSR II benchtop analyzer for flow cytometry. The data were gated to calculate the percentage of nucleated cells of each marker type in the sample and the following two combinations of markers named MSC1 and MSC2. The first definition was CD90^+^, CD44^+^, and CD45^−^ (MSC1). This was designed to be a broad definition of possible MSCs. The second definition was more stringent and included CD90^+^, CD44^+^, CD45^−^, CD14^−^, and CD34^−^ (MSC2).

### Fluorescent-Activated Cell Sorting and Differentiation

The remaining cells were cultured in T-75 flasks with growth medium (DMEM with 10% FBS and 1% PEST) until confluence. Plastic adherent cells were lifted and sorted using fluorescent-activated cell sorting (FACS) to get a viable, homogenous population of CD90^+^, CD44^+^, and CD45^−^ cells. Cells were placed in 50 ml tubes with DNase I solution. Ten milliliters of PBS was slowly added dropwise while swirling the tube, then centrifuged at 500 × *g* for 5 min, and the supernatant was discarded. The cells were washed and resuspended in 1 ml PBS with dextrose + 1 mM EDTA. Cells were counted using Trypan blue staining. Antibodies for CD90, CD45, and CD44 were added to the tubes and incubated. The cells were sorted into the defined populations using FACS such that a pure population with the appropriate cell marker profile was produced.

The cells were plated in six-well plates (1.8 × 10^4^ cells/well) and cultured in growth medium until 90% confluence prior to differentiation. For chondrogenic differentiation, cells were lifted, washed with PBS, and resuspended in DMEM. Four conical tubes were seeded with 2 × 10^5^ cells and centrifuged generating a pellet. The supernatant was replaced with growth medium (control; *n* = 2) or chondrogenic medium (treatment; *n* = 2). The chondrogenic medium (10 ng/ml DMEM with high glucose, 10 ng/ml TGF-B3, 4 mM l-glutamine, 10^−7^ M dexamethasone, 0.17 mM ascorbic acid, 0.35 mM l-proline, 1 mM sodium pyruvate, 1% PEST) was changed every 2–3 days for 3 weeks (24). The pellets were sectioned and stained with Alcian Blue for glycosaminoglycan.

For osteogenic differentiation, the medium in two wells was replaced with osteogenic medium (DMEM, 10% FBS, 10 mM β-glycerophosphate, 10^−7^ dexamethasone, and 0.05 mM ascorbic acid-2-phosphate, 1% PEST) (25). Two additional wells were maintained with growth medium as controls. The osteogenic medium was changed every 3 days for 3 weeks and the staining was performed on the cells with Alizarin Red. A subjective comparison between control and differentiated samples was used to determine positive and negative staining.

### Statistical Analysis

Nucleated cell concentration (nucleated cell/g of tissue or ml of blood), total nucleated cell count, and potential MSCs using both definitions were calculated for each site and dog. The total number of potential MSCs was calculated by multiplying the percentage of cells expressing CD90^+^, CD44^+^, and CD45^−^ (MSC1) or CD90^+^, CD44^+^, CD45^−^, CD14^−^, and CD34^−^ (MSC2) in the sample by the total number of nucleated cells.

Matched pairs *t*-tests were used to statistically compare sites. Variation was compared among sites using a Levine test. Patient body condition was also evaluated with respect to MSC numbers using regression analysis. Differences were considered significant at *P* < 0.05.

## Results

### Harvest

All 10 dogs were under 2 years of age with a range of 13–21 months old. The median body condition score was 4.5 with a range of 3.5–7 out of 9. Patient body weight and body condition score were not significantly associated with total cell concentration. The mean volume of bone marrow samples collected was 20 ml (range 10.5–27 ml). The mean weight of all adipose tissue samples collected was 29 g (range 14–55 g). All falciform fat samples were 30 g or higher, but harvesting 30 g of subcutaneous fat was not always possible despite extensive dissection. The harvest of bone marrow overall was slightly more challenging compared to the adipose given the strength and aim necessary to get through the cortex and into the marrow. The ilium was more difficult than the humerus or tibia. The easiest site to harvest was the falciform.

### Flow Cytometry

The data for each biomarker, site, and tissue harvested are presented in detail in Tables 1 and 2. Table 1 contains the mean and SD of total cells by marker, and Table 2 gives the total nucleated cells, the nucleated cell concentration, total potential MSCs for each tissue, and the potential MSCs for each tissue as a percent of the total nucleated cell count.

**Table 1:** **Mean (±SD) percentage of cells for each marker and site of collection**.

Site of collection (mean% of cells **±** SD)					
Marker	Humerus	Ilium	Tibia	Falciform	SQ shoulder
CD44^+^	26.78 ± 2.64	21.87 ± 3.48	19.35 ± 2.24	49.05 ± 5.24	43.25 ± 3.02
CD14^−^	66.42 ± 3.00	69.37 ± 4.07	73.25 ± 2.91	55.78 ± 4.43	54.50 ± 2.15
CD90^+^	25.21 ± 2.41	25.17 ± 2.21	21.69 ± 2.11	31.75 ± 3.23	36.28 ± 3.22
CD166^+^	51.55 ± 1.78	49.43 ± 3.38	51.50 ± 2.93	32.76 ± 3.67	47.74 ± 3.91
CD45^−^	63.13 ± 3.18	61.97 ± 6.83	70.42 ± 3.27	68.43 ± 2.10	62.63 ± 2.75
CD34^−^	61.13 ± 1.96	61.97 ± 2.95	2.93 ± 2.39	48.08 ± 2.12	32.02 ± 6.28

**Table 2:** **Numbers and concentrations of cells**.

Site of collection (mean **±** SD)MSC 1 = CD90^+^ CD44^+^ CD45^−^MSC 2 = CD90^+^ CD44^+^ CD45^−^ CD14^−^ CD34^−^					
	Humerus	Ilium	Tibia	Falciform	SQ shoulder
Total nucleated cell number (×10^6^)	851.63 ± 108.54	966.85 ± 321.89	131.99 ± 55.86	12.60 ± 1.40	4.93 ± 0.91
Nucleated cell concentration (cells × 10^6^/ml bone marrow or cells × 10^5^/g adipose)	39.25 ± 4.25	23.10 ± 1.97	4.50 ± 1.00	3.72 ± 0.45	2.10 ± 0.33
Total MSCs MSC 1 (×10^6^)	1366.66 ± 360.59	1348.12 ± 823.05	78.91 ± 27.07	82.86 ± 19.42	25.25 ± 4.16
Total MSCs MSC 2 (×10^6^)	120.90 ± 25.22	584.54 ± 473.64	17.25 ± 7.01	5.10 ± 1.36	0.73 ± 0.19
MSC 1% of nucleated cells	1.50 ± 0.35	1.08 ± 0.24	0.69 ± 0.11	7.71 ± 2.71	5.55 ± 0.84
MSC 2% of nucleated cells	0.13 ± 0.02	0.32 ± 0.15	0.19 ± 0.05	0.44 ± 0.14	0.23 ± 0.09

*Mean (±SD) of total nucleated cell number, concentration of nucleated cells, total number of potential mesenchymal stem cells (MSCs), and MSCs based on definition MSC 1 and MSC 2 as a percentage of nucleated cells*.

**Figure 1:**
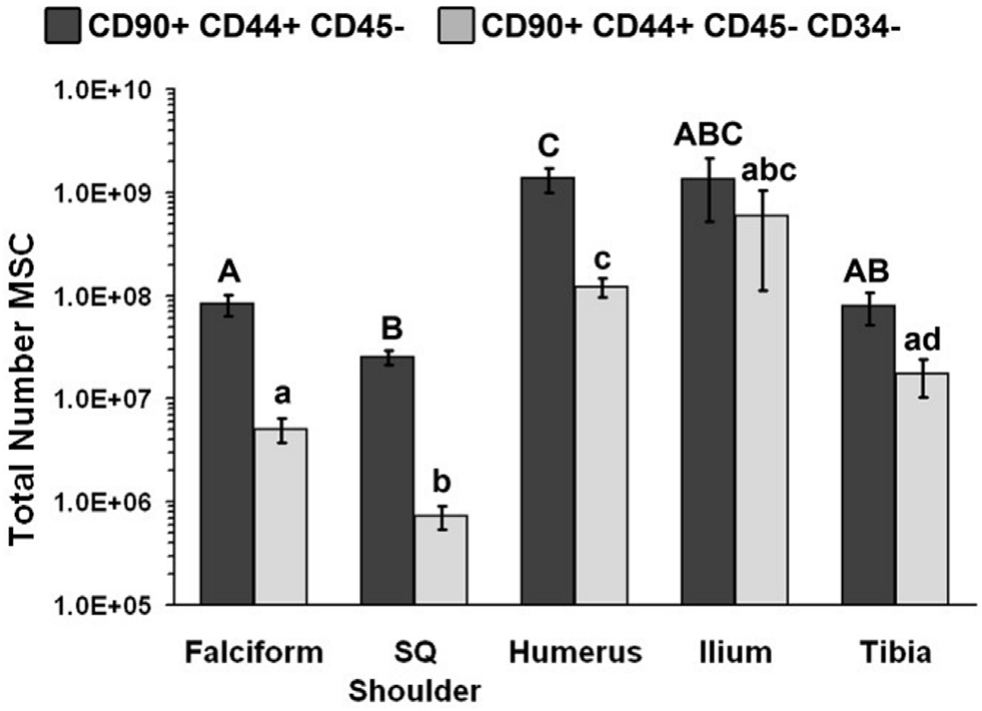
**Total number of MSCs collected at each site (mean ± SD)**. Cells were designated as MSCs based on one of the two definitions: definition 1: CD90^+^, CD44^+^, and CD45^−^ (A–C); definition 2: CD90^+^, CD44^+^, CD45^−^, CD14^−^, and CD34^−^ (a–d). Different letters above bars indicate significant (*P* < 0.05) differences among sites. In other words, those labeled with the same letter are not statistically different. Upper case letters indicate differences based on the analyses of three cell markers, whereas lower case letters indicate differences between analyses of five cell markers. Graph has been log transformed.

The nucleated cell concentrations harvested were significantly (*P* < 0.05) increased in aspirates harvested from the humerus when compared to the ilium and tibia. Similarly, the nucleated cell concentrations were significantly (*P* < 0.05) increased in adipose tissue harvested from the falciform ligament when compared to the caudal scapular region. The total nucleated cell counts obtained from the humerus and the ilium were significantly higher (*P* < 0.05) when compared to the total cell numbers harvested from the tibia, falciform ligament, and the caudal scapular subcutaneous region (Figure 1, Table 2).

**Figure 2:**
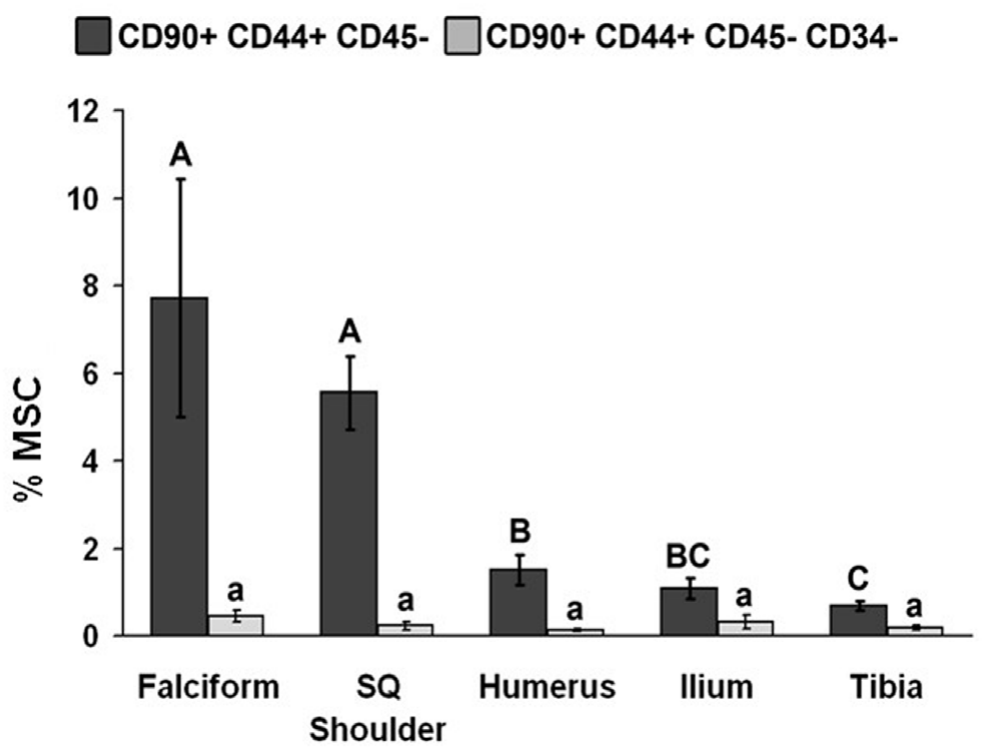
**Percentage of MSCs from each extraction site (mean ± SD)**. Cells were designated as MSCs based on one of the two definitions: definition 1: CD90^+^, CD44^+^, and CD45^−^ (A–C); definition 2: CD90^+^, CD44^+^, CD45^−^, CD14^−^, and CD34^−^ (a–d). Different letters above bars indicate significant (*P* < 0.05) differences among sites. In other words, those labeled with the same letter are not statistically different. Upper case letters indicate differences based on the analyses of three cell markers, whereas lower case letters indicate differences between analyses of five cell markers. Graph has been log transformed.

The potential MSC concentration from the falciform and subcutaneous shoulder adipose was significantly higher (*P* < 0.05) when compared to the humerus, ilium, and tibia using cell surface markers CD90^+^, CD44^+^, and CD45^−^ (Figure 2). However, the total number of potential MSCs isolated from the humerus was significantly (*P* < 0.05) higher when comparing those isolated from the tibia, falciform ligament, and the caudal scapular subcutaneous region using cell surface markers CD90^+^, CD44^+^, and CD45^−^ (Figure 1). When using the more stringent MSC definition, the pattern remained similar for each site but statistical differences were not always significant. Variation was limited in the adipose tissue samples compared to bone marrow (*P* < 0.05).

### FACS and Differentiation

**Figure 3:**
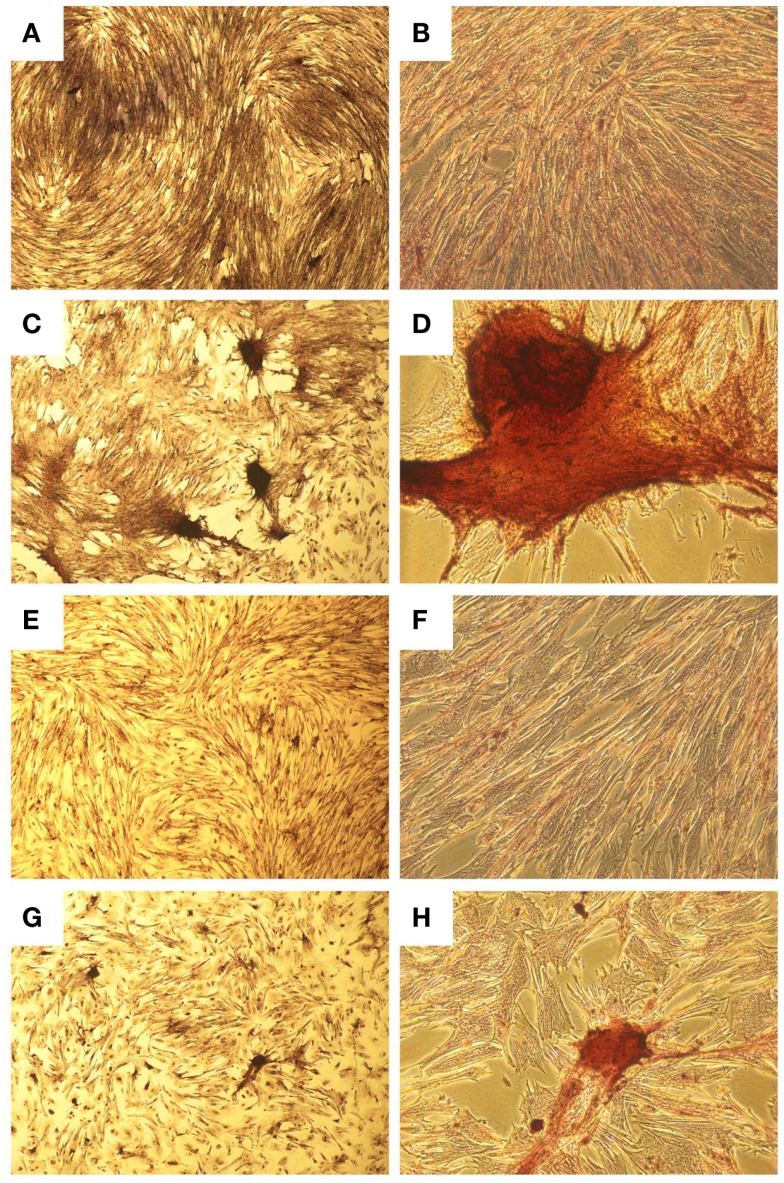
**Histological images showing osteogenic differentiation of cells**. Adipose tissue **(A–D)** or bone marrow **(E–H)** stained with Alizarin Red for calcium at two magnifications. Control aMSCs [**(A)** 4×; **(B)** 20×] or differentiated aMSCs in osteogenic medium [**(C)** 4×; **(D)** 20×]. Control bMSCs [**(E)** 4×; **(F)** 20×] or differentiated bMSCs in osteogenic medium [**(G)** 4×; **(H)** 20×].

**Figure 4:**
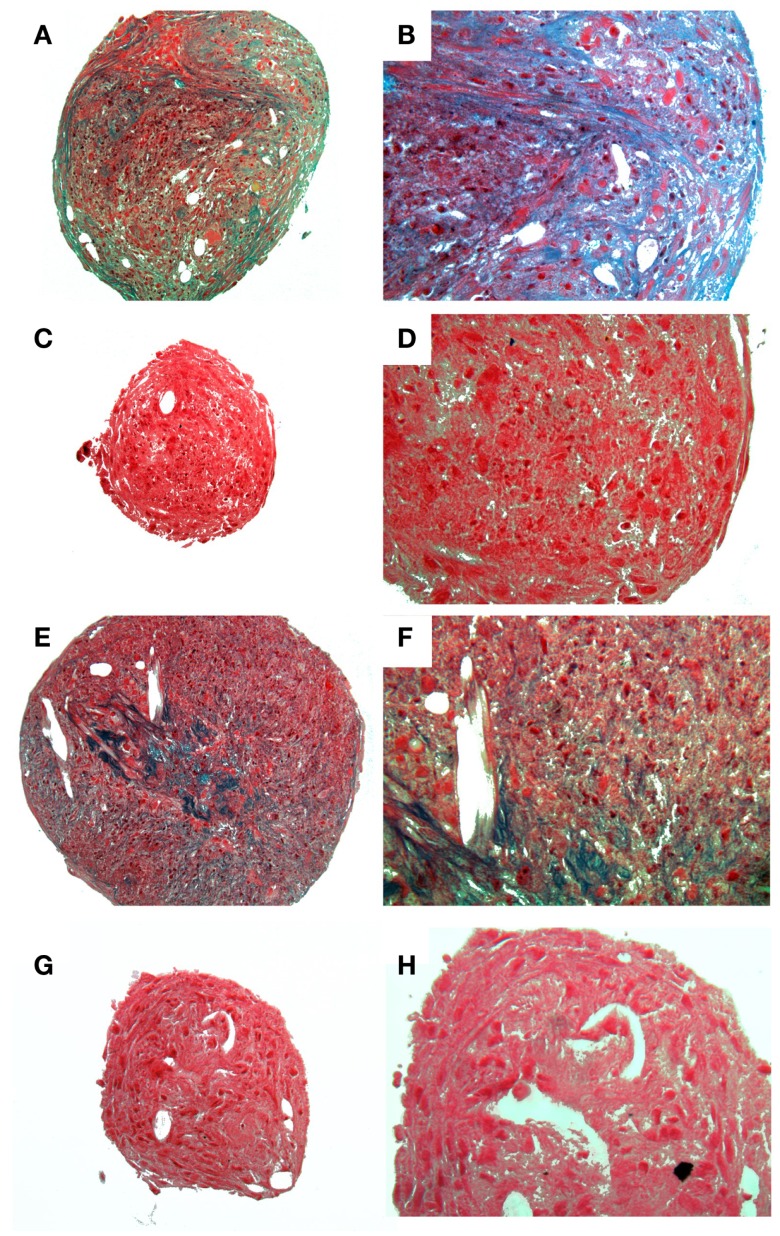
**Histological images showing chondrogenic differentiation**. Adipose tissue **(A–D)** or bone marrow **(E–F)** at two magnifications stained for glycosaminoglycans with Alcian Blue. Control aMSCs [**(A)** 4×; **(B)** 20×] or differentiated aMSCs in chondrogenic medium [**(C)** 4×; **(D)** 20×]. Control bMSCs [**(E)** 4×; **(F)** 20×] or differentiated bMSCs in chondrogenic medium [**(G)** 4×; **(H)** 20×].

A homogenous population of CD90^+^, CD44^+^, and CD45^−^ cells adhered to plastic and proliferated to 90% confluence. Subjective evaluation of the cells treated with osteogenic media showed positive Alizarin Red staining over the cells subjected to growth media. The pelleted cells subjected to chondrogenic media showed subjectively greater glycosaminoglycan deposition than control pellets as stained by Alcian Blue (Figures 3 and 4). No other testing was performed to further elucidate or quantify differentiation efficiency or character.

## Discussion

### Harvest

Clinically, the technical difficulty of the harvest is very important. It is arguable which tissue harvest is more invasive. All methods of harvest had some degree of morbidity. Harvesting bone marrow was technically more difficult than harvesting adipose tissue, which is consistent with our first hypothesis. For adipose tissue collection, the caudal scapular subcutaneous tissue region required more dissection and did not yield as much tissue as the falciform fat region. This was likely related to the generally low body condition of the dogs. Clinical populations of dogs may have higher body condition scores and thus more subcutaneous adipose deposits. Other sites may yield different or better results (i.e., inguinal fat) (26, 27). Among the bone marrow sites, the humerus was subjectively the easiest site to sample, followed by the tibia, and the ilium was the most difficult site for bone marrow harvesting.

Human adipose tissue is more readily abundant and accessible than human bone marrow (28). Our results may differ due to a lower ratio of fat to muscle in the population of canines used when compared to humans. The great abundance and ease of adipose tissue harvest in humans, where samples are commonly obtained in liters after liposuction, are far greater than in dogs where 30 g may be difficult to obtain (2). Interestingly, in this study, body condition was not related to the total number of potential MSCs in adipose tissue. This is likely due to the limited range of body condition in the study population.

### Characterization of Cell Populations

Ideally, the content of a solution advocated for the treatment of any disease would be known with the least amount of variation possible. SVF has been advocated for several diseases in dogs, but the content of the SVF has been extrapolated from other species. This study describes a step in defining the SVF in dogs. Similar to humans, our research shows that adipose tissue has a higher percentage of potential MSCs (as defined by the biomarkers used) than bone marrow (2, 16). However, we must reject our second hypothesis as bone marrow had a higher total nucleated cell count than adipose tissue. This is likely because of the numbers of white blood cells expected in bone marrow but absent in adipose tissue.

Even with a higher concentration of potential MSCs in the SVF, there are still 92.3% of cells that are undefined in dogs and injected into joints or tissues. In humans, SVF is made up of white blood cells (25–45%), endothelial cells (10–20%), pericytes (3–5%), and stromal cells (15–30%) (4).

No individual site was best for all outcomes assessed (i.e., total number of nucleated cells, total number of potential MSCs, cells/tissue collected, variability, etc.). The best site varied depending upon which outcome was assessed. For instance, the tibia yielded lower total number of potential MSCs, but it was also the most consistent. Fat from the falciform ligament contained a greater concentration of cells defined as MSC1, which is consistent with our third hypothesis. However, bone marrow-derived potential MSC1 cells were harvested in greater total number. This makes determining the best location for stem cell harvest difficult. Some outcomes are more important than others depending on the potential use of the MSCs. SVF injected into a joint or tendon would ideally have high number of MSCs and a low number of other nucleated cells (high percentage of MSCs), assuming that the remaining nucleated cell population is not therapeutic and that the minimum number of MSCs required is present. However, if the SVF was going into culture, the highest total starting number is arguably more important.

Cell yields vary between collection sites and species (18, 21, 29–31). Specifically in dogs, Neupane et al. found that inguinal fat had a low number of cells per gram of fat, and that cells did not proliferate well after adhering to plastic (18). Subcutaneous fat appeared most promising, followed by omental fat (18). Even though the study presented here and Neupane calculated the concentration of MSCs harvested from subcutaneous fat, the flow cytometery was performed on adherent cells rather than the SVF (18). This eliminated non-adherent cell populations, preventing direct comparison between studies. Perhaps more importantly, some studies address the differences between sites with regard to differentiation postharvest (30, 32). The ability to differentiate was assessed in this study, but the efficiency or quality of differentiation was not compared among the sites or tissues. These cell populations and their markers may be different from the SVF that is currently being injected back into patients (8, 16).

The data in this study must be evaluated in light of the limitations. There is no definitive set of markers defining MSCs for the dog in either cultured MSCs or the SVF (6, 15). Some studies report clear differences among various species with respect to expression of markers (3, 33, 34). In this study, the markers chosen were identified based on consistency across other species and known reactivity of the antibodies to canine cells. Thus, it is possible that other markers would provide a more definitive profile of canine MSCs. However, a recent study evaluated canine MSCs derived from bone marrow, adipose tissue, muscle, and periosteum with similar markers, documenting expression of CD44 and 90 but not of 34 and 45 (35). Bourin et al. proposed a set of markers to standardize the evaluation of SVF for humans, including CD90^+^, CD44^+^, CD73^+^, CD13^+^, CD29^+^, CD105^+^, CD45^−^, CD235^−^, CD31^−^, and CD34^+^ (4). The MSC1 definition used in this article completely overlaps but is broader than that proposed characterization. Based on these criteria, it can be argued that we included populations of cells other than MSCs in our comparison. Using the MSC2 definition, which was more stringent and CD34^−^ (in contrast to Bourin et al.), the number of MSCs was low. It is possible that dogs have less concentrated MSC populations or that different markers are needed for this species. The presence or absence of CD34 as a marker for MSCs is controversial in dogs and is affected by the harvest technique and amount of hemorrhage (4, 6, 15). The amount of hemorrhage was not specifically quantified in these dogs, although the surgical technique was standardized. Ideally, we would have tested all known markers for MSCs and the subsequent differentiation to develop an ideal canine formula, which may even have varied between bone marrow and adipose tissue. This would not be practical given the anticanine antibodies and fluorochromes available at the time.

The label of MSC has been assigned based on different criteria in the literature from the simple (i.e., adherence of cells to plastic) to the more robust (i.e., specific marker profiles, proliferation rates, and differentiation into fat, cartilage, bone, and more) (5, 9, 10, 30, 36–41). This study presents some evidence of differentiation, but it is not as robust as would be argued by Dominici et al. For instance, Dominici et al. proposed that differentiation into adipose tissue was necessary to determine the differentiation potential of MSCs (5). Additionally, further characterization of the tissue by PCR, immunohistochemistry, or other biochemical techniques would have provided additional evidence of differentiation rather than the staining techniques used here. Using this definition, we could not call the cells in this study MSCs. However, if the simplest definition in the literature (plastic adherent cells) is used, the cells isolated with the given markers would be considered MSCs despite the chance that some of the sorted cells are progenitor cells rather than MSCs (9, 10, 30, 32, 36–40). Most studies assessing the biomarker profile of MSCs use adherent cells for differentiation rather than a specific population of cells sorted for the biomarker profile. Although differentiation is tested in these studies, a mixed population of cells is often used, making it difficult to establish differentiation of the cells with the phenotype presented or the remaining population of cells (1, 2, 7, 10, 16, 18, 20, 25, 28, 32, 38, 40). Progenitor cells may be part of the population driving some of the differentiation. Using the second more stringent definition of MSC (MSC2) may be eliminating progenitor cells.

Vieira et al. also performed extensive outcome measures for differentiation of cultured canine MSCs including flow cytometry, proliferation of plastic adherent cells, histopathologic staining, and PCR (42). Although the differentiation data are weak in this study, the markers used are consistent with those shown and differentiated by Vieira et al. (42). The aMSCs from dogs were characterized by flow cytometry for the expression of 10 cell surface proteins (CD13, CD29, CD31, CD34, CD44, CD45, CD73, CD90, CD105, and CD117). At passage 4, the majority of adipose derived stem cells (cASCs) expressed CD44, CD29 (β1 integrin), and CD90 (Thy1) adhesion molecules. Other markers, including CD14, CD34, CD45, and CD117, were consistently absent in few cells (42). This shows that the expression characteristics change or the cell population changes in plastic adherent cells, thus injecting plastic adherent cells into a joint or tendon may be different than injecting the SVF.

The SVFs were frozen before flow cytometry in our study. This may have affected the expression of cell surface markers or the viability of the cells. However, one study showed that the proliferation and differentiation capacity as well as the cellular characteristics were identical in freshly derived bone marrow-derived stem cells (bMSCs) and bMSCs derived after freezing and storage (43).

The variation in our data among dogs was large. This degree of variation is consistent with previous studies in other species (34). MSC cell yield had also been shown to be quite variable among patients in several studies and may be affected by several factors, including harvesting site or method (38). The volume obtained while harvesting the marrow was variable despite the use of a standardized needle and syringe for all sites. It is possible that when a large volume was obtained, the sample was hemodiluted adding to the variability of the bone marrow samples. One reason for very small samples may have been random needle occlusion either by clot or tissue. This information was not recorded at the time of harvest.

Age and weight may also contribute to variation. One study showed that attachment and proliferation capacity are more pronounced in aMSCs derived from younger donors compared with older donors (44, 45). The dogs in this study were all skeletally mature, but they were also young (<2 years). Therefore, results in older dogs may differ. This is substantiated by a study reporting decreasing nucleated cells in the SVF derived from adipose in successive age groups (26).

Although a correlation with body condition was not found in this study, it is possible that more aMSCs could be procured from canine subjects with greater body condition scores. This study did not include obese dogs. The mean body condition score of dogs sampled in this study was 4.5, which is quite lean. However, one human study showed that the ratio of adipocytes to MSCs is constant in humans, independent of body mass index and age (46). This allows a greater harvest of MSCs from patients or subjects with a higher BMI.

Based on the surgical and *in vitro* data presented, fat from the falciform ligament appears to be the logical choice to harvest tissue, if the SVF is going to be injected back into the injury site. Although bone marrow yielded the greatest total number of CD90^+^, CD44^+^, and CD45^−^ cells, the falciform ligament was the easiest to harvest, provided the most consistent yield, and possessed the highest ratio of CD90^+^, CD44^+^, and CD45^−^ cells to other nucleated cells. This research may be used as a foundation for future research and development of cell-based therapies in the dog.

## Author Contributions

MS participated in funding, study design, all data collection and analysis, and manuscript preparation. WG-E participated in funding, study design, all data collection and analysis, and manuscript preparation. LF participated in study design, technical assistance with antibody identification and preparation, FACs, flow cytometry, cell storage and handling, differentiation, and manuscript preparation. KK participated in study design, provided the dogs, and participated in sample collection from the dogs and processing prior to cryopreservation. She also participated in manuscript preparation. MC participated in study design, provided dogs, and participated in sample collection, provided laboratory facilities, equipment, and advice. He also participated in manuscript preparation. DG participated in study design, troubleshooting problems during the study, manuscript preparation and editing, and provided laboratory facilities and equipment. All authors approved the final manuscript.

## Conflict of Interest Statement

The authors declare that the research was conducted in the absence of any commercial or financial relationships that could be construed as a potential conflict of interest.

## Footnotes

^1^AbD Serotec Raleigh, NC, USA.

^2^Lynx Rapid Ab Conjugation Kit, AbD Serotec.

## Abbreviations

aMSCs, adipose-derived mesenchymal stem cells; bMSCs, bone marrow-derived stem cells; DMEM, Dulbecco’s Modified Eagle’s Medium; FACS, fluorescent-activated cell sorting; MSCs, mesenchymal stem cells; PEST, penicillin/streptomycin.
